# A Miniature Binocular Endoscope with Local Feature Matching and Stereo Matching for 3D Measurement and 3D Reconstruction

**DOI:** 10.3390/s18072243

**Published:** 2018-07-12

**Authors:** Di Wang, Hua Liu, Xiang Cheng

**Affiliations:** 1 Department of Instrument Science and Engineering, SEIEE, Shanghai Jiao Tong University, Shanghai 200240, China; wangdi_z@sjtu.edu.cn (D.W.); touch1995@sjtu.edu.cn (X.C.); 2 Shanghai Engineering Research Center for Intelligent Diagnosis and Treatment Instrument, Shanghai 200240, China

**Keywords:** endoscope, binocular stereoscopic vision, local feature descriptor, stereo matching, 3D measurement, 3D reconstruction

## Abstract

As the traditional single camera endoscope can only provide clear images without 3D measurement and 3D reconstruction, a miniature binocular endoscope based on the principle of binocular stereoscopic vision to implement 3D measurement and 3D reconstruction in tight and restricted spaces is presented. In order to realize the exact matching of points of interest in the left and right images, a novel construction method of the weighted orthogonal-symmetric local binary pattern (WOS-LBP) descriptor is presented. Then a stereo matching algorithm based on Gaussian-weighted AD-Census transform and improved cross-based adaptive regions is studied to realize 3D reconstruction for real scenes. In the algorithm, we adjust determination criterions of adaptive regions for edge and discontinuous areas in particular and as well extract mismatched pixels caused by occlusion through image entropy and region-growing algorithm. This paper develops a binocular endoscope with an external diameter of 3.17 mm and the above algorithms are applied in it. The endoscope contains two CMOS cameras and four fiber optics for illumination. Three conclusions are drawn from experiments: (1) the proposed descriptor has good rotation invariance, distinctiveness and robustness to light change as well as noises; (2) the proposed stereo matching algorithm has a mean relative error of 8.48% for Middlebury standard pairs of images and compared with several classical stereo matching algorithms, our algorithm performs better in edge and discontinuous areas; (3) the mean relative error of length measurement is 3.22%, and the endoscope can be utilized to measure and reconstruct real scenes effectively.

## 1. Introduction

The endoscope is a new nondestructive testing instrument which focuses on traditional optics, ergonomics, precision machinery, modern electronics, mathematics and software [1], which can enlarge the range of vision and horizon and improve the resolution of observation. It is able to work in restricted spaces as well as in high temperature, high pressure, toxic and harmful environments. In the industrial field, it can accurately and clearly detect the internal situation of machinery such as jet engines and automobiles without disassembling them. In the medical field, doctors can use endoscopes to observe the position and size of internal wounds in the body and perform minimally invasive surgery. To some extent, the endoscope can help doctors judge the severity of the disease quickly and efficiently, which also can improve the accuracy of diagnoses.

With the development of 3D measurement technology, 3D measurement and 3D reconstruction are becoming more significant in the industrial and medical field. The 3D endoscope has become a hot topic. In 2012, Choi et al. proposed an endoscope with a miniature flipping glass disk, which can measure the size of objects using several known optical and geometrical parameters, [2]. In 2014, Guo et al. proposed a new type of binocular optical system with a dual objective lens and a single image sensor and the measurement error of the system is within ±0.2 mm [3]. The endoscope’s probe has an outside diameter of 6 mm. In 2016, Furukawa et al. designed an endoscope with a Diffractive Optical Element (DOE) projector inside. They also proposed a new line-based grid pattern with gap coding to successfully reconstruct the shape of the soft tissue of a human subject [4].

At present, the Olympus (Shinjuku, Tokyo, Japan) and Everest VIT (Piaseczno k. Warszawy, Poland) endoscopes are leading the world and almost monopolize the high-end electronic endoscope market. Both the probes of Mentor Visual iQ^TM^ and IPLEX NX have a diameter of 4 mm with a resolution of 440,000 pixel [5,6]. Besides, with the capability of measuring length, depth, perimeter and area, they are highly-priced. The endoscopes of several representative companies in China such as Shenyang Shenda Endoscope Co. Ltd. (Shenyang, China) and Shanghai Aohua Endoscopy Co. Ltd. (Shanghai, China) have a diameter of 1 mm to 10 mm without the capability of stereo measurement [7,8]. Recently, Beijing Dellon Inspection Technology Co., Ltd. (Beijing, China) and Xvzhou TENOSI Visual Technology Co. Ltd. (Xvzhou, China) have individually introduced an industrial endoscope equipped with stereo measurement capability [9,10], but these two endoscopes have diameters of 6 mm and 8 mm, respectively, making them too large to access restricted spaces.

In this paper, a 3D endoscope whose probe has a maximum diameter of 3.17 mm and contains two CMOS cameras with a resolution of 160,000 pixels is developed. The size of the endoscope is far smaller than the sizes of other binocular endoscopes, which makes it possess better ability to access confined spaces to detect and measure objects. In the usage of endoscopes, the matching accuracy of left and right pixels directly influences the measurement precision. Therefore, a construction method of the WOS-LBP descriptor which satisfies actual requirements of endoscopes is proposed and applied in the designed endoscope to match pixels concisely as well as achieve 3D measurement. Besides, the endoscope can utilize a stereo matching algorithm proposed in this paper to obtain disparity maps of images taken by it. Then 3D point clouds data can be acquired from the disparity maps by triangulation to realize 3D reconstruction for real scenes. With the proposed descriptor and the stereo matching algorithm, the endoscope can effectively achieve 3D measurement and 3D reconstruction in tight and confined spaces.

## 2. Methods

### 2.1. System Principle and Composition

#### 2.1.1. Principle of Binocular Stereo Vision

Because of the interpupillary distance (approximately 65 mm for adults) when observing distant objects, the field angle of the eyes varies for objects at different distances from the same perspective. It is known from the projection relationship of geometrical optics that points at different distances from the observers are imaged at different locations on the retina, and this difference in position is called binocular parallax. Binocular parallax reflects the depth of objects and gives a sense of stereoscopic vision [11].

**Figure 1:**
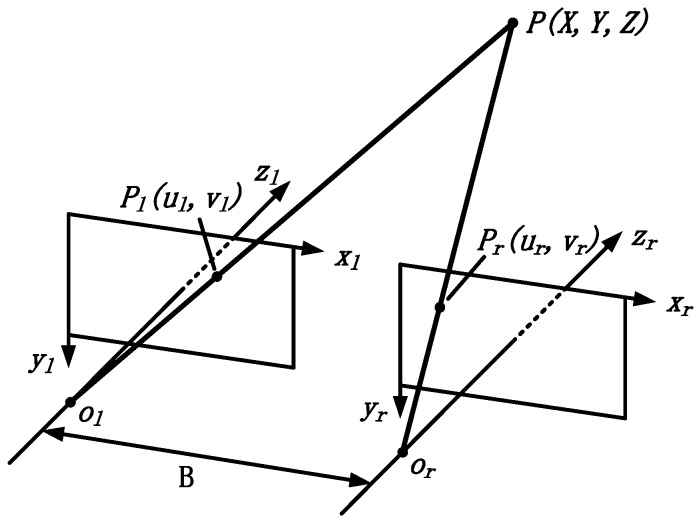
Principle of binocular stereo imaging.

Two CMOS cameras with the same parameters are used to simulate the eyes. The principle of binocular stereoscopic vision is shown in Figure 1. The two CMOS cameras have exactly the same focal length as well as intrinsic parameters and they are coplanar. The *x*-axes of each camera mutually coincidee and the *y*-axes are parallel to each other. The *z*-axes (optical axis) of each camera are parallel to each other and both are perpendicular to the camera. The distance between the two cameras’ optical centers (B) is called the baseline distance. Assuming the spatial coordinate point P(X,Y,Z) is imaged at $P_{l}\left(u_{l},v_{l}\right)$ on the left image and at $P_{r}\left(u_{r},v_{r}\right)$ on the right image, and the two imaging pixels are on the same line, which means $v_{l}=v_{r}$. It can be obtained from the principle of triangular measurement that:

(1) $$\{\begin{array}{l} u_{l}=f\frac{X}{Z}\\ u_{r}=f\frac{X-B}{Z}\\ v_{l}=v_{r}=f\frac{Y}{Z} \end{array}$$

where f is the focal length of the two cameras. The disparity can be deduced by:

(2) $$d=u_{l}-u_{r}=f\frac{B}{Z}$$

so, the 3D coordinates can be obtained:

(3) $$\{\begin{array}{c} X=\frac{Bu_{l}}{d}\\ Y=\frac{Bv_{l}}{d}\\ Z=\frac{fB}{d} \end{array}$$

Equation (2) represents the fundamental principal of binocular stereoscopic imaging, whereby the disparity is inversely proportional to the depth, namely, the further away the object is, the smaller the disparity. 

#### 2.1.2. Structure of Probe

**Figure 2:**
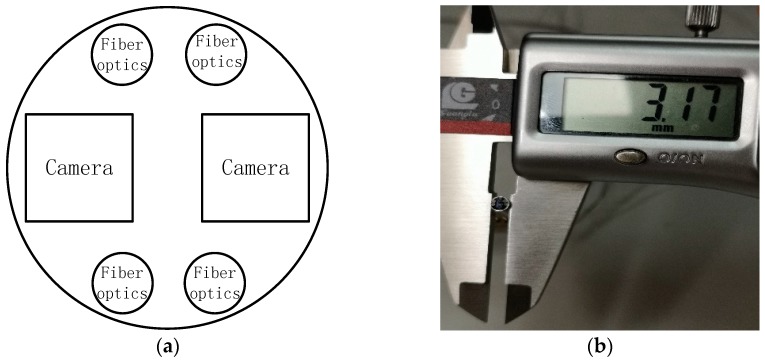
(**a**) Schematic diagram of endoscopic head; (**b**) Prototype of endoscopic head.

Two CMOS cameras with a resolution of 400 × 400 pixels and package dimensions of 1.05 mm × 1.05 mm × 2.266 mm are chosen as the image sensors of the designed probe. As shown in Figure 2, the probe contains two cameras and four fiber optics for illumination, while, its dimension is kept to be 3.17 mm in diameter. The distance between the two optical centers of the two cameras is designed to be 1.6 mm.

### 2.2. Local Feature Descriptor

The basic premise of 3D measurement is to find corresponding pixels in the left and right images. Therefore, matching of points of interest (POI) is an important step in 3D measurement and can be achieved by local feature descriptors. POI mentioned in this paper refer to any pixel selected by the operator, rather than pixels with some special characteristics, because in the process of 3D measurement, pixels to be measured are selected by the operator instead of being detected automatically. Local feature descriptors usually fall into four categories: the distribution-based descriptor, the filter-based descriptor, the moment-based descriptor and the binary descriptor. Among the four descriptors, the distribution-based descriptors show the best performance, including SIFT [12], PCA-SIFT [13], SURF [14] and etc., but they are complicated to construct. In practical usage of binocular endoscopes, a slight relative rotation generates between the two cameras, while different noises and illumination may exist in the images. Thus, local feature descriptors must have high performance in rotation invariance, noise resistance, robustness to illumination as well as short construction time to achieve precise and quick matching of POI. Conclusively, the binary descriptor fulfills all the requirements above with simple construction method. So, based on the binary descriptor, a construction method of the WOS-LBP descriptor is proposed and applied in the endoscope.

#### 2.2.1. Main Orientation Extraction

In order to achieve the rotation invariance of local feature descriptors, it is necessary to obtain the main orientation of POI. Select a POI-centric circular area of 16 × 16 pixel, calculate the gradient magnitude and gradient direction of the pixels in the circular area:

(4) $$m\left(x,y\right)=\sqrt{{\left(I\left(x+1,y\right)-I\left(x-1,y\right)\right)}^{2}+{\left(I\left(x,y+1\right)-I\left(x,y-1\right)\right)}^{2}}$$

(5) $$\theta \left(x,y\right)=tan^{-1}\left(I\left(x,y+1\right)-I\left(x,y-1\right)/I\left(x+1,y\right)-I\left(x-1,y\right)\right)$$

where I(x,y) is the gray value of (x,y).

**Figure 3:**
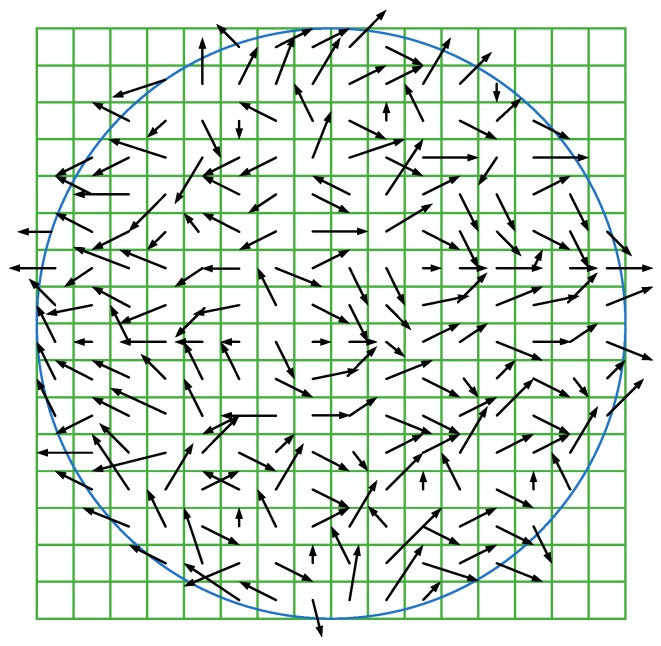
Gradient magnitude and direction of pixels in a circular area.

The gradient direction of every pixel in the circular area is recorded by an orientation histogram which has 36 bins covering the 360 degrees range of orientations. Add the gradient magnitude of every pixel in the circular area to the orientation histogram corresponding to its gradient direction, the highest peak in the histogram represents the main orientation of the POI. The extraction schematic is shown in Figure 3.

#### 2.2.2. Construction and Matching of the Local Feature Descriptor

**Figure 4:**
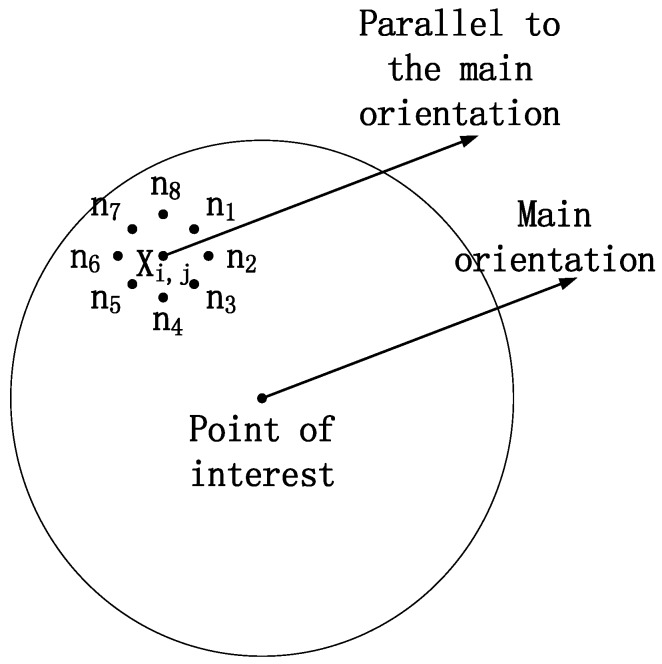
The encoding order of neighborhood pixels.

Local binary pattern [15] (LBP) has good rotation and gray-scale invariance, which is robust to illumination and simple to construct. Because of these advantages, LBP is often used to construct the local feature descriptors of POI. However, LBP only considers the intensity relationship between the POI and every neighborhood pixel, and the dimension of LBP descriptors (256 dimensions) is too high; while the proposed WOS-LBP descriptor can improve distinctiveness and robustness of local features by comparing gray value of neighborhood pixels which are orthogonal-symmetric with respect to the center pixel as well as reduce the size of descriptors. The construction of the WOS-LBP descriptor can be divided into three steps:

- Step 1: determine the encoding order of neighborhood pixels. For any pixel *X_i,j_* in the feature region of POI, calculate the angles between the main direction and the lines joining the neighborhood pixels and the POI respectively. Then establish the direction coordinate system of *X_i,j_* and select the neighborhood pixel with the smallest angle as *n*_1_ as well as the encoding start pixel. Finally encode the remaining neighborhood pixels clockwise as *n*_2_, *n*_3_, *n*_4_, *n*_5_, *n*_6_, *n*_7_, *n*_8_ (see Figure 4).
- Step 2: Add the four neighborhood pixels which are orthogonal-symmetry with respect to *X_i,j_* into one group, then two 4-orthogonal-symmetric groups can be obtained, namely [*n*_1_, *n*_3_, *n*_5_, *n*_7_] and [*n*_2_, *n*_4_, *n*_6_, *n*_8_]. Encode the two groups:
  (6) $$WOS-LBP_{1,8}^{\left(m\right)}\left(X_{i,j}\right)=sign\left(n_{i}-n_{i+4}\right)2^{0}+sign\left(n_{i+2}-n_{i+6}\right)2^{1}$$
  with:
  $$sign\left(x\right)=\{\begin{array}{c} 1,x>T\\ 0,other \end{array},\;m=1,2,\;T=2$$
  The result of the Equation (6) can be 0, 1, 2 and 3 which is written as 0001, 0010, 0100 and 1000 respectively.
- Step 3: Two 4-dimension feature descriptors can be obtained after the above two processes. Connect the two descriptors in series, then an 8-dimension WOS-LBP descriptor can be acquired.

For example, suppose the gray value of *X_i,j_* is 128 and the gray value from *n*_1_ to *n*_8_ is 125, 123, 127, 128, 130, 129, 126, 122. Then the bit string of [*n*_1_, *n*_3_, *n*_5_, *n*_7_] is 0001 and the bit string of [*n*_2_, *n*_4_, *n*_6_, *n*_8_] is 0100. Connect the two bit strings in series and the WOS-LBP descriptor of *X_i,j_* is 00,010,100. Through processes above, the WOS-LBP descriptor of a single pixel can be obtained. However, it’s likely to result in mismatch by a feature descriptor of a single pixel because of the low distinctiveness. Thus, combine the constructing idea of the distribution-based descriptor with the WOS-LBP descriptors, divide the feature region of POI into several subregions and do a statistical survey of local feature descriptors in each subregion, which can be described as follows:

- Step 1: Select a circular area centered on the POI with a radius of 8 pixels as the feature region, then divide the feature region into five subregions from the center to the outside and ensure the numbers of pixels within each subregion are similar.
- Step 2: Extract R, G, B color components from the color information of the feature region, calculate the WOS-LBP descriptors of all the pixels in each subregion *K_l_* of every color component, then construct a distributed histogram to record the obtained feature descriptors in which each bin indicates each dimension of the feature descriptors. The calculation equation is:
  (7) $$H^{m}\left(k\right)=\underset{X_{i,j}\in K_{l}}{\sum }f(WOS-LBP_{1,8}^{\left(m\right)}\left(X_{i,j}\right),k)$$
  (8) f(x,y)={exp(−(i2+j2)2∗(1.5σ)2),x=y0,x≠y
  where *l* = 1, 2, 3, 4, 5, *m* = 1, 2, *k* = 0, 1, 2, 3, *i* and *j* is the relative offset of a pixel *X_i,j_* in the subregion towards the POI, σ is a constant, normally 1.6.
- Step 3: Arrange the local feature descriptors of every color component in each subregion by relevant rules to finally generate the local feature descriptors of the POI.

The dimensions of a WOS-LBP descriptor are as low as 8 × 3 × 5 = 120, compared with SIFT descriptors (128-dimension), CS-LBP descriptors (256-dimension) and LBP descriptors (512-dimension). Assuming the WOS-LBP descriptor of a reference pixel is *L*(*x*_1_, *x*_2_, …, *x*_120_) and the WOS-LBP descriptor of a waiting-for-matched pixel is *R*(*y*_1_, *y*_2_, …, *y*_120_), then the calculation equation of Euclidean distance between the two pixels is:

(9) $$D=\sqrt{\sum_{i=1}^{120}{\left(x_{i}-y_{i}\right)}^{2}}.$$

Calculate the Euclidean distance between the reference pixel and every waiting-for-matched pixel with Equation (9), then choose the waiting-for-matched pixel with minimal distance as the matching pixel. The disparity of the two pixels and three-dimensional coordinate of spatial point P(X,Y,Z) can be derived from Equations (2) and (3). Three-dimensional measurement of objects can be deduced by three-dimensional coordinates of multiple points.

### 2.3. Stereo Matching

Stereo matching is one of the research focuses in computer vision, at its key ideal is to construct three-dimensional models for space scenes by matching the pixels in multiple images from different perspectives point-by-point and seeking for the three-dimensional coordinate of space points afterwards. Stereo matching is divided into four steps: cost initialization, cost aggregation, disparity computation and disparity optimization. And existing stereo matching algorithms can be categorized by global method and local method. The global method minimizes an energy function to obtain the optimal matching pixel while the local method overlays the pixel-value differencing of pixels in a local window. As the local method is advantageous in computation time and implementation, most of the stereo matching algorithms conduct similarity metric measuring based on pixels’ luminance or gray value, e.g., absolute intensity differences (AD), squared intensity differences (SD), adaptive weight, the Census transform [16], etc. It is proved by Hirschmuller et al. that the method for acquiring matching cost based on the Census transform is robust to light distortion [17]. Zhang et al. constructed a cross-based adaptive region based on the color differences of pixels [18].

In terms of the advantages and disadvantages of current algorithms a stereo matching algorithm is proposed based on Gaussian-weighted AD-Census transform and improved cross-based adaptive regions; disparity optimization algorithm based on vote method, information entropy and region growing algorithm is adopted for the optimization of unreliable pixels’ disparities.

#### 2.3.1. Cost Initialization

Traditional Census transformation (CT) compares gray value of a pixel with its neighborhood pixels’ gray value to generate a bit string, then the matching cost of two pixels is calculated by Hamming distance. For a pixel *p*, the Census transformation is:

(10) $$CT\left(p\right)={⊗}_{q\in W\left(p\right)}\zeta \left(I\left(p\right),I\left(q\right)\right)$$

(11) $$\zeta \left(I\left(p\right),I\left(q\right)\right)=\{\begin{array}{l} 1,I\left(p\right)<I\left(q\right)\\ 0,other \end{array}$$

where *W*(*p*) is the neighborhood window of *p* which has a gray value of *I*(*p*), and *q* is pixels in *W*(*p*). CT only considers the gray relationship between *p* and each of its neighborhood pixels and ignores the position relationship. Thus, the pixel value of *p* is replaced by the Gaussian-weighted value in this paper, and the equation is:

(12) $$I_{wm}=\left[\frac{1}{D}\sum_{q\in W\left(p\right)}W_{pq}I_{q}\right]$$

(13) Wpq=exp−(x2+y2)2σ2

where *I_wm_* is the weighted pixel value, $D=\sum W_{pq}$ is the sum of weights, *x* and *y* is the position offset of the pixels in the window towards the center pixel *p*, and σ is a standard deviation of 1.5.

Assuming that *p*(*x*, *y*) is a pixel in the left image, *q*(*x − d*, *y*) is the correspondent pixel in the right image, and *d* is the disparity of the two pixels. Taking two separate costs *C_census_*(*p*, *d*) and *C_AD_*(*p*, *d*) into consideration, *C_AD_* is the sum of the absolute values of color differences among *R*, *G*, *B* components between the two pixels, and *C_census_* is defined as the Hamming distance of the Census strings of them:

(14) $$C_{AD}\left(p,d\right)=\sum_{i=R,G,B}\left|I_{i}^{Left}\left(p\right)-I_{i}^{Right}\left(q\right)\right|$$

(15) $$C_{census}\left(p,d\right)=Hamming\left(CT\left(p\right),CT\left(q\right)\right)$$

Combine the two costs above, the total cost function is:

(16) $$C\left(p,d\right)=\rho \left(C_{census}\left(p,d\right),\gamma_{census}\right)+\rho \left(C_{AD}\left(p,d\right),\gamma_{AD}\right)$$

where ρ(c,γ) is a robust function on variable c:

(17) $$\rho \left(c,\gamma \right)=1-exp\left(-\frac{c}{\gamma }\right)$$

#### 2.3.2. Improved Cross-Based Local Support Region Construction

In stereo matching, the optimal matching pixels are retained by comparing the similarities between the reference pixel in the reference image and every waiting-for-matched pixel in the searching range of maximum disparity in another image. Meanwhile, it’s easy to occur mismatch because of the low distinctiveness of a single pixel. So, the window in an appropriate size for similarity matching should be created to improve the distinctiveness.

**Figure 5:**
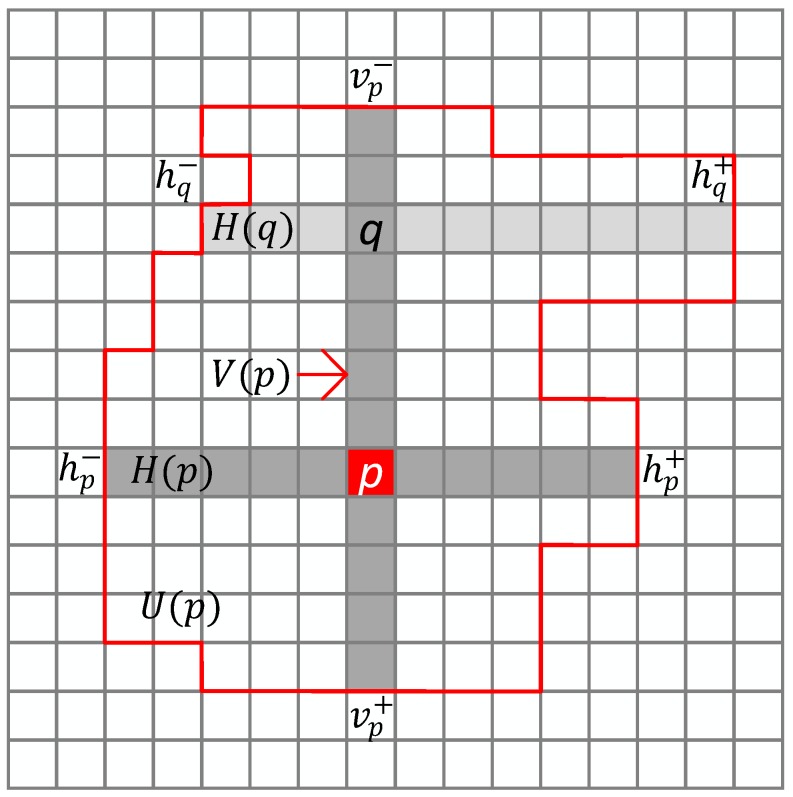
Cross-based local support window.

Utilizing the assumption that pixels with similar intensity within a constrained area are likely from the same image structure and have similar disparity, Zhang [18] put forward a method for constructing a cross-base local support region for each pixel. For the anchor pixel p, construct a cross-based region composed by the horizontal segment *H*(*p*) and the vertical segment *V*(*p*) as the initial local support skeleton (see Figure 5). The size of the cross-based region is determined by $\left\{h_{p}^{-},h_{p}^{+},v_{p}^{-},v_{p}^{+}\right\}$ which denotes the left, right, up, bottom arm length respectively. The determination criteria of the arm length are:

1. $D_{c}\left(p_{i},p\right)<\tau$;
2. $D_{d}\left(p_{i},p\right)<L$;

In criterion 1, $D_{c}\left(p_{i},p\right)=\underset{c\in \left\{R,G,B\right\}}{max}(\left|I_{c}\left(p_{i}\right)-I_{c}\left(p\right)\right|)$ is defined as the maximum value of the color difference between p and $p_{i}$; in criterion 2, $D_{d}\left(p_{i},p\right)$ is the space distance of the two pixels, which limits the maximum value of the arm length and avoids excessive growth of the local window. τ and *L* is the preset color and distance threshold. This method only takes into account the color differences between every regional pixel and the anchor pixel and is lack of consideration in the color differences of adjacent pixels. On this basis, the criterions are improved by increasing a gradient threshold for adjacent pixels and two different color thresholds in this paper. The gradient value is computed by Scharr operator [19] here. The optimized criteria are:

1. $D_{c}\left(p_{i},p\right)<\tau_{1},\;if\;D_{d}\left(p_{i},p\right)<L_{1}$;
2. $D_{c}\left(p_{i},p\right)<\tau_{2},\;if\;L_{1}<D_{d}\left(p_{i},p\right)<L_{2}$;
3. $D_{d}\left(p_{i},p\right)<L_{2}$;
4. $D_{G}\left(p_{i},p_{i}+\left(1,0\right)\right)<\beta_{1}$

where $\tau_{1}$ and $\tau_{2}$ are two different color thresholds ($\tau_{1}>\tau_{2},\;\tau_{2}=0.5\times \tau_{1}$), $L_{1}$ and $L_{2}$ are two different distance thresholds ($L_{2}>L_{1},\;L_{1}=0.5\times L_{2}$), and $\beta_{1}$ is a gradient threshold for adjacent pixels. For textureless areas, when the distance exceeds $L_{1}$, a large window can be acquired while the excessive growth of the window is avoided by using the smaller threshold $\tau_{2}$. For textured areas, the larger color gradient $\tau_{1}$ is used to retain the window in an appropriate size.

**Figure 6:**
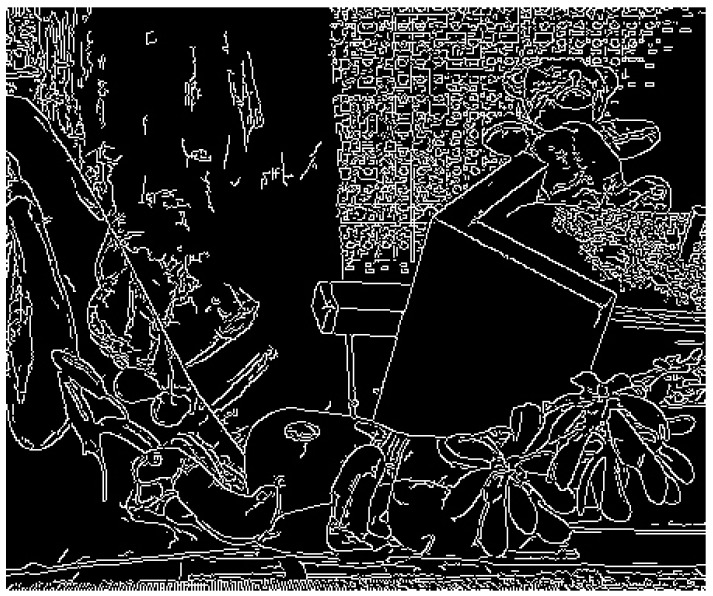
The edge and discontinuous areas.

For edge and discontinuous areas, adjustments for thresholds are required to further reduce the growth of windows. Canny operator [20] is used to filter out the edge and discontinuous areas as the white areas shown in Figure 6. For these areas, the adjusting criteria are given here:

1. $\tau_{1}^{\prime }=0.75\times \tau_{1},\;\tau_{2}^{\prime }=0.5\times \tau_{1}^{\prime },\;L_{2}^{\prime }=0.5\times L_{2},\;L_{1}^{\prime }=0.5\times L_{2}^{\prime },\;if\;G\left(p\right)>\beta_{2}$;
2. thresholds do not change, $if\;G\left(p\right)\leq \beta_{2}$;

The four arm lengths $\left\{h_{p}^{-},h_{p}^{+},v_{p}^{-},v_{p}^{+}\right\}$ for the pixel p can be confirmed by the improved criterion, and then the horizontal segment *H*(*p*) and the vertical segment *V*(*p*) can be acquired:

(18) $$\{\begin{array}{l} H\left(p\right)=\left\{\left(x,y\right)|x\in \left[x_{p}-h_{p}^{-},x_{p}+h_{p}^{+}\right],y=y_{p}\right\}\\ V\left(p\right)=\left\{\left(x,y\right)|x=x_{p},y\in \left[y_{p}-v_{p}^{-},y_{p}+v_{p}^{+}\right]\right\} \end{array}$$

Zhang models the local support region by integrating multiple horizontal segments *H*(*q*), sliding along the vertical segment *V*(*p*) of the pixel *p*, which only considers the differences among horizontal pixels and ignores the differences among vertical pixels. On this basis, the local region is constructed by jointing two windows, one of the windows is modeled by Zhang’s method and another is modeled by integrating multiple vertical segments *V*(*q*) sliding along the horizontal segment *H*(*p*) of the pixel *p*:

(19) $$U\left(p\right)=\left(\cup_{q\in V\left(p\right)}H\left(q\right)\right)\cup \left(\cup_{q\in H\left(p\right)}V\left(q\right)\right).$$

#### 2.3.3. Cost Aggregation

Select the pixel p(x,y) in the left image as the reference pixel and the pixel q(x−d,y) in the right image as the waiting-for-matched pixel. The disparity of the two pixels is *d*. Two adaptive windows U(p) of p(x,y) and $U^{\prime }\left(q\right)$ of q(x−d,y) can be generated by the improved adaptive window method proposed in the previous section. The overlaps of the two windows are assigned as the final joint region:

(20) $$U_{d}\left(p\right)=\left\{\left(x,y\right)|\left(x,y\right)\in U\left(p\right),\left(x-d,y\right)\in U^{\prime }\left(q\right)\right\}.$$

**Figure 7:**
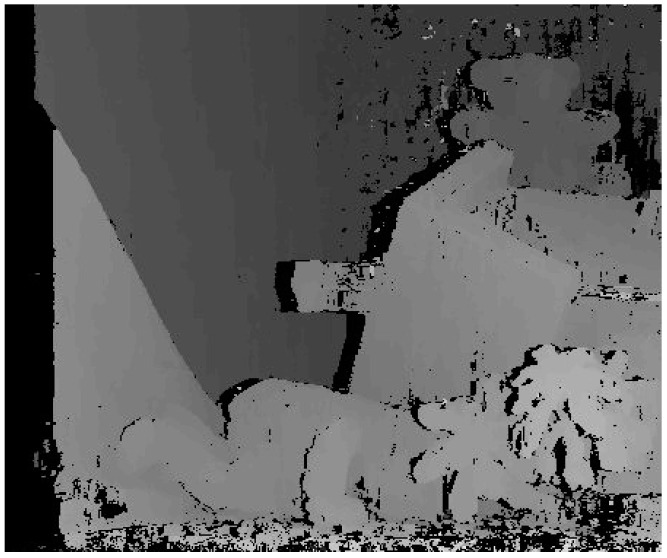
The initial disparity map.

Aggregate the matching costs of all the pixels in $U_{d}\left(p\right)$ by Equations (14)–(17) to obtain the final matching cost:

(21) $$E\left(p,d\right)=\frac{1}{N}\sum_{s\in U_{d}\left(p\right)}C\left(s,d\right)$$

where *s* represents the pixels in $U_{d}\left(p\right)$ and *N* represents the number of *s*. Finally, take Winner-Takes-All (WTA) method to determine the initial disparity of every pixel. The initial disparity map is shown in Figure 7.

#### 2.3.4. Disparity Optimization

There are mismatches in the matching process, which lead to the deviation between the initial disparity and the real disparity. Therefore, the initial disparity reliability should be verified and the wrong disparity needs to be optimized.

Left-Right-Differences (LRD) is adopted to verify the initial disparity reliability and the specific method is implemented as follows: choose the left image as the reference image while the right one is the waiting-for-matched image to get one disparity map as $D_{1}$, and then reverse this process to get another disparity map as $D_{2}$. It is assumed that the disparity of the pixel p(x,y) in $D_{1}$ is $d_{1}$ and the disparity of the pixel $q\left(x-d_{1},y\right)$ in $D_{2}$ is $d_{2}$. The initial disparity reliability of p(x,y) can be verified by the formula:

(22) $$\left|d_{1}-d_{2}\right|\leq Th$$

where Th is a preset threshold. If the difference between $d_{1}$ and $d_{2}$ is greater than Th, p(x,y) is considered to be invalid and its disparity is unreliable. Otherwise, p(x,y) is considered to be valid and its disparity is reliable.

For every unreliable disparities, an optimization method based on region voting is proposed. Suppose p(x,y) is an invalid pixel. Then, count up number of all pixels as *N* and number of valid pixels as Votes in the cross-based region of p(x,y). And according to Votes and *N*, there are three conditions for optimization:

- Condition 1: If Votes<N/3, search for the nearest valid pixel from p(x,y) to the left and right and replace the disparity of p(x,y) with the disparity of the valid pixel found. If no valid pixels are found, search to the top and bottom.
- Condition 2: If N/3≤Votes<2×N/3, the average value of the disparities of all the valid pixels in the region is calculated as the optimized disparity of p(x,y).
- Condition 3: If 2×N/3≤Votes, construct a disparity histogram and replace the disparity of p(x,y) with the value of the bin with the highest peak.

**Figure 8:**
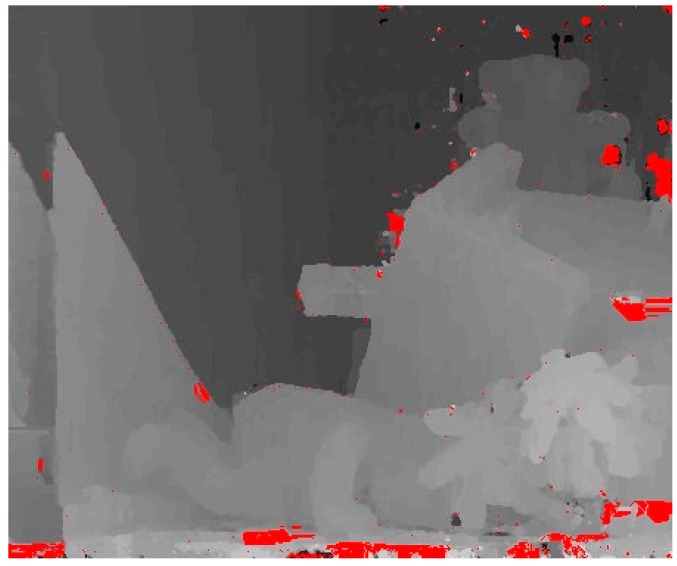
The disparity map after the first optimization.

Finally, the optimized invalid pixels are set as valid pixels. The disparity optimization method can be performed on all invalid pixels effectively and most of disparities can be ensured to be reliable. Nevertheless, there may be mismatches caused by occlusion which result that the initial disparities deviated from the real disparities are considered to be reliable by LRD and can’t be optimized by the proposed disparity optimization method. For parts of the pixels with these disparities shown in Figure 8 (the red areas), a method based on image entropy and region-growing algorithm is proposed to extract them and optimize their disparities.

Image entropy is a kind of information entropy which represents geometric average of the image grayscale and its magnitude shows the intensity of the change in pixels [21]. The calculation equation of one-dimensional image entropy is as follows:

(23) $$E=\sum_{i=0}^{255}p_{ij}logp_{ij}$$

where $p_{ij}$ represents the appearance probabilities of gray value in a local window. Set the pixels whose entropy is less than 0.5 as invalid pixels and expend the regions around these pixels by region-growing algorithm. The algorithm is as follows:

- Step 1: Choose one pixel from the invalid pixels as an initial pixel $\left(x_{0},y_{0}\right)$ each time.
- Step 2: Search the neighborhood pixels (x,y) of $\left(x_{0},y_{0}\right)$. If $\left|I\left(x_{0},y_{0}\right)-I\left(x,y\right)\right|\leq 3$ and (x,y) hasn’t been searched, set (x,y) as another initial pixel.
- Step 3: Repeat the Step 2 until all the initial pixels has been searched, then a consecutive region is obtained. If the number of the pixels in the region is smaller than 2000, set all the pixels in the region as invalid pixels. Otherwise, set them as valid pixels.
- Step 4: Repeat Step 1 until all the invalid pixels has been chosen.

**Figure 9:**
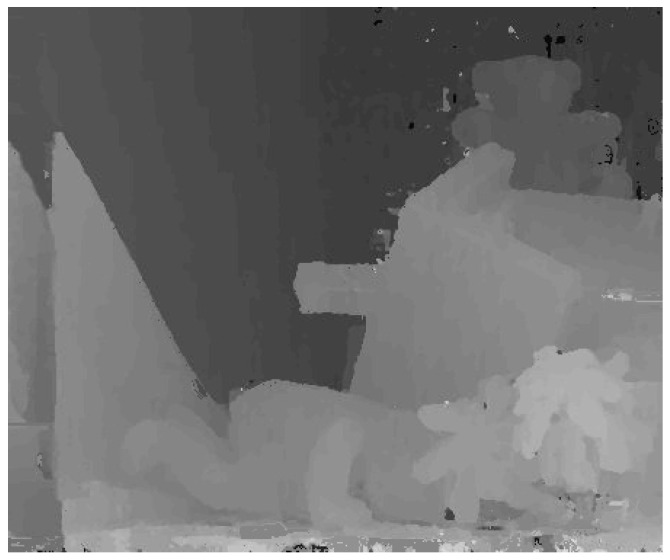
The disparity map after the second optimization.

For the invalid pixels obtained by region-growing algorithm, the proposed disparity optimization method is adopted to optimize their disparities. As shown in Figure 9, disparities of most of mismatched pixels are effectively optimized.

## 3. Experiments and Results

Several simulation experiments were carried out on the WOS-LBP descriptor and the proposed stereo matching algorithm. The designed endoscope was used to carry out tests for length measure and 3D reconstruction for real scenes. The simulation platform is a PC equipped with a 2.50 GHz Intel (R) Core (TM) i5-7300HQ CPU (Lenovo, Beijing, China). In addition, the proposed adopts Visual Studio 2010 (Microsoft Corporation, Redmond, WA, USA), Matlab 2015b (The Mathworks, Natick, MA, USA) as the experimental platform.

### 3.1. Evaluation of Local Feature Descriptor

**Figure 10:**
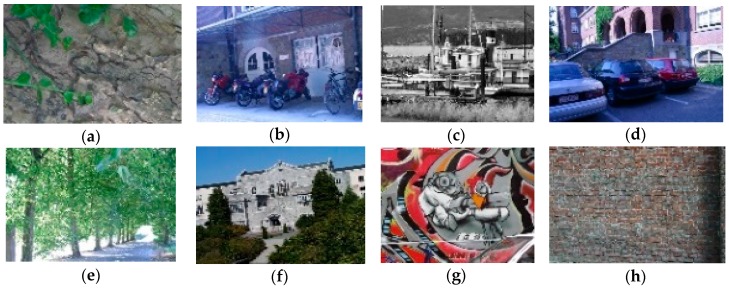
(**a**) Bar; (**b**) Bikes; (**c**) Boat; (**d**) Leuven; (**e**) Tree; (**f**) Ubc; (**g**) Wall1; (**h**) Wall2.

The WOS-LBP descriptor is tested in its rotation invariance, noise resistance, robustness to illumination and construction time. In order to give fair and objective evaluations of descriptors, the Oxford Data Set [22] is chosen as the test set. The images in the test set are shown in Figure 10. Other local feature descriptors for comparison are: LBP [15], CS-LBP [23], SIFT [12] and SURF [14] among which LBP and CS-LBP descriptors are constructed by the region segmentation approach described in this paper.

10,000 pixels were randomly selected in each test image and the average construction times of the five descriptors of these pixels were calculated respectively. The test results are shown in Table 1.

We can see from the results that the average construction time of the WOS-LBP descriptor is almost equal to that of the CS-LBP descriptor, and is half of that of the LBP descriptor. And the average construction time of the SIFT descriptor is about five times more than that of the WOS-LBP descriptor, because the SIFT descriptor is obtained in DOG (Difference of Gaussians) scale-space and its construction algorithm has high complexity; while, the average construction time of the SURF descriptor is far less than that of other descriptors, because integral image is used for acceleration in the process of construction. Compared with the CS-LBP descriptor, the LBP descriptor and the SIFT descriptor, the WOS-LBP descriptor is more compact and spends less time in construction. Methods are adopted to test the rotation invariance and distinctiveness of the local feature descriptors:

- Step 1: Create three other rotating image sets by rotating each image in the test set by 90 degrees, 180 degrees and 270 degrees respectively.
- Step 2: Randomly, assign 1000 pixels in every image without rotation as the reference pixels.
- Step 3: In every rotating image set, search the matched pixels through the 5 local feature descriptors respectively. The coordinates of the obtained pixels should exactly match that of the reference pixels if successful.

**Figure 11:**
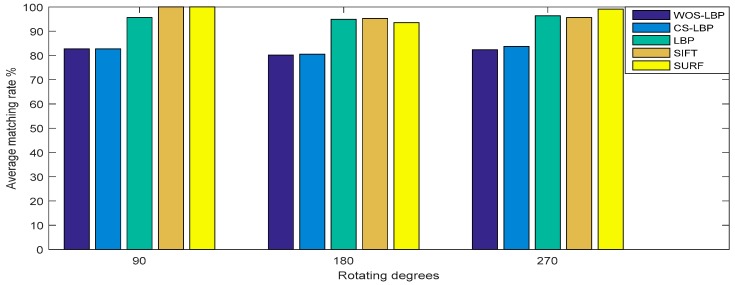
Test results of rotation invariance and distinctiveness.

Test results are shown in Figure 11.

It can be seen from Figure 11 that the average matching rates of LBP, SIFT and SURF are at about 96%, while the matching rates of WOS-LBP and CS-LBP are slightly lower and at a rate about 82%. It shows that all of the five descriptors have excellent performance regarding rotation invariance and distinctiveness as their matching rates have exceeded 80%.

**Figure 12:**
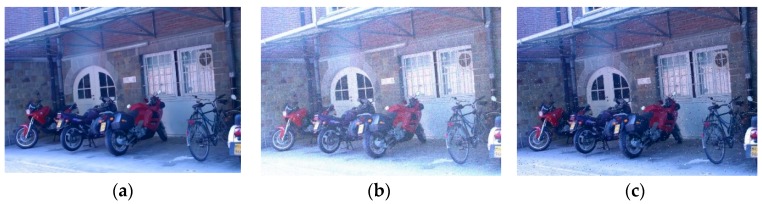
(**a**) Original image; (**b**) Image with Gaussian noise; (**c**) Image with salt-and-pepper noise.

Gaussian noise and salt-and-pepper noise were added into every image in the original test set respectively to obtain two other test sets, which were utilized to test the noise resistance of the five descriptors. Portions of the images in the three test sets are shown in Figure 12. Test results are shown in Table 2.

From Table 2, we can find that the first three descriptors have preferable robustness to salt-and-pepper noise, while performing unfavorable robustness to Gaussian noise for the reason that their constructions by using pixel value directly are sensitive to Gaussian noise. Meanwhile, gradient information and Haar wavelet transform are used in the construction process of SIFT descriptors and SURF descriptors respectively, which make them display almost robustness towards salt-and-pepper noise and Gaussian noise.

**Figure 13:**
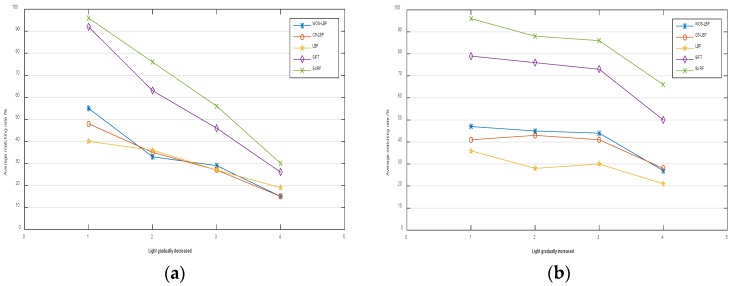
(**a**) The result of decreasing light; (**b**) The result of increasing light.

In order to test the robustness towards the light change of the five descriptors, the image named bikes in the test set was selected and its light intensity was changed to generate another 10 images with different light intensities. The results are shown in Figure 13.

The robustness to light change decreases with the increase of illumination distortion. The SIFT descriptor and the SURF descriptor have better robustness than other three descriptors and the WOS-LBP descriptor and CS-LBP descriptor perform slightly better robustness than the LBP descriptor.

In general, the proposed WOS-LBP descriptor has the advantages of good rotation invariance, short construction time and low size while ensuring reasonable distinctiveness and robustness, which indicates that it can be applied in the endoscope for 3D measurement. Compared with the LBP descriptor and the CS-LBP descriptor, the WOS-LBP descriptor is more compact, which is to say that it has lower size while having the same descriptive ability as the other descriptors.

### 3.2. Evaluation of Stereo Matching

Middlebury standard pairs of images were utilized to test our algorithm. The four pairs of images are: Cones, Teddy, Tsukuba and Venus. The search ranges are 0~59, 0~59, 0~15 and 0~19 pixels, respectively. The parameters in our algorithm are shown in Table 3.

**Figure 14:**
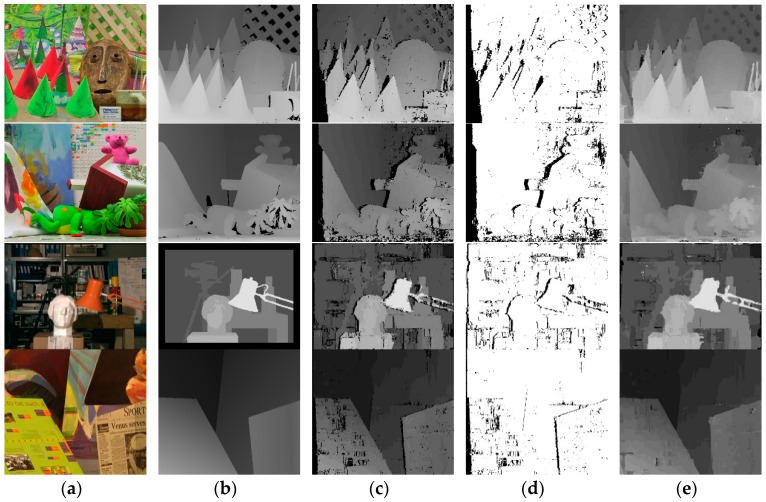
(**a**) Left reference view; (**b**) Ground truth; (**c**) Initial disparity map; (**d**) Invalid pixels (black areas); (**e**) Final disparity map.

The disparity results are presented in Figure 14. Table 4 shows the percentage of bad matching pixels whose absolute disparity error is greater than 1. The experimental results show that the proposed algorithm is better than SNCC [24] and its average percentage of bad matching pixels is almost equal to that of GC + occ [25] and ESAW [26], while it performs better than GC + occ and ESAW in the texture images of Teddy and Cones because of the proposed optimization on edge and discontinues areas. Compared with SemiGlob [27], our algorithm is a little higher in the average percentage of bad matching pixels but it is lower in the percentage of bad matching pixels in edge and discontinuous areas.

**Figure 15:**
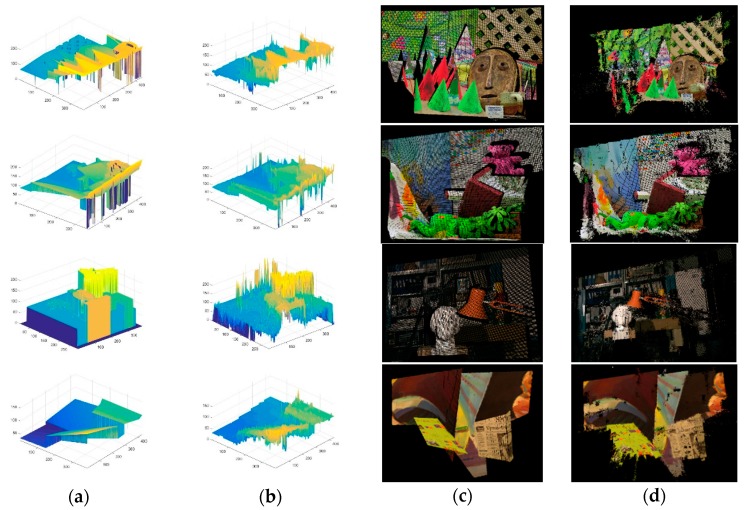
(**a**) 3D display for ground truth; (**b**) 3D display for final disparity map; (**c**) 3D reconstruction for ground truth; (**d**) 3D reconstruction for final disparity map.

In addition, through the obtained disparity map, 3D coordinate of every pixel in the image can be obtained by Equation (3) with the known f and B. Therefore, 3D displays for disparity maps and 3D point clouds data can be carried out on Matlab 2015b and Visual Studio 2010 with Point Cloud Library (PCL), which are shown in Figure 15.

### 3.3. Length Measurement and 3D Reconstruction Using the Designed Endoscope

Before using the binocular endoscope, the calibration method proposed by Zhengyou Zhang [28] and Bouguet’s algorithm [29] were adopted to calibrate and rectify the cameras. The calibration template is a chessboard which has 12 × 9 squares, the side length of every square is 1.5 mm. Results of camera calibration are shown in Table 5.

From the two intrinsic matrixes we can find that the focal lengths of the two cameras are nearly equal and the coordinate of every optical center is also near the theoretical value which is (200, 200). Besides, the rotation matrix is close to an identity matrix of 3-order which is to say only slight relative rotation generates between the two cameras. The above indicates that the parameters of the two cameras are similar and the two cameras satisfy the basic requirement of the binocular vision. We choose the camera coordinate of the left camera as the world coordinate, so f=252.0886 pixel/mm and B=−1.5976 mm.

**Figure 16:**
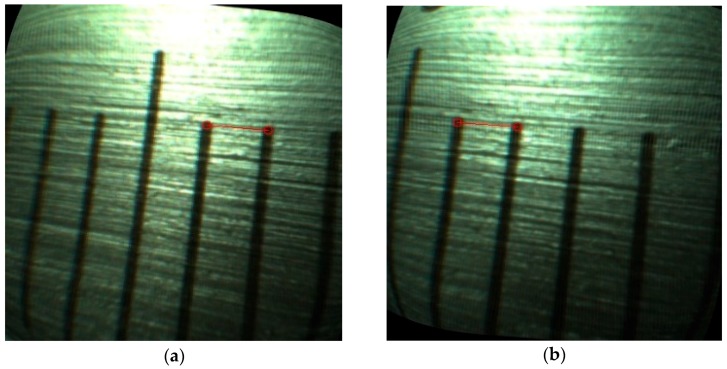
(**a**) Left view; (**b**) Right view.

To evaluate the endoscope under realistic conditions, several experiments of measuring the scale of a Vernier caliper from different depths were carried out with the designed endoscope. The measured line is the red line shown in Figure 16. The ends of the line in the left view are selected by the operator and the match of them adopts the WOS-LBP descriptor. From Figure 16, it is indicated that the ends of the line in the left view can be well matched in another view. The disparities and 3D coordinates of the two endpoints can be obtained by Equations (2) and (3). Then the length of the line can be measured. The standard length of the measured line is 1 mm and test results are shown in Table 6.

It can be observed from Table 6 that the maximum error is 0.0801 mm and the mean relative error of the measurement is 3.22%, which shows the endoscope can be used for 3D measurement effectively.

**Figure 17:**
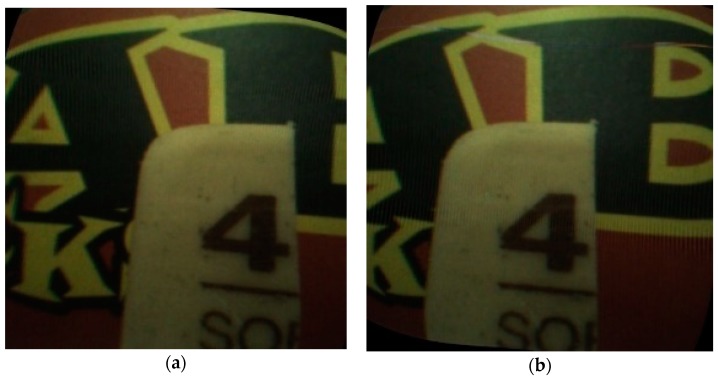
(**a**) Left view; (**b**) Right view.

**Figure 18:**
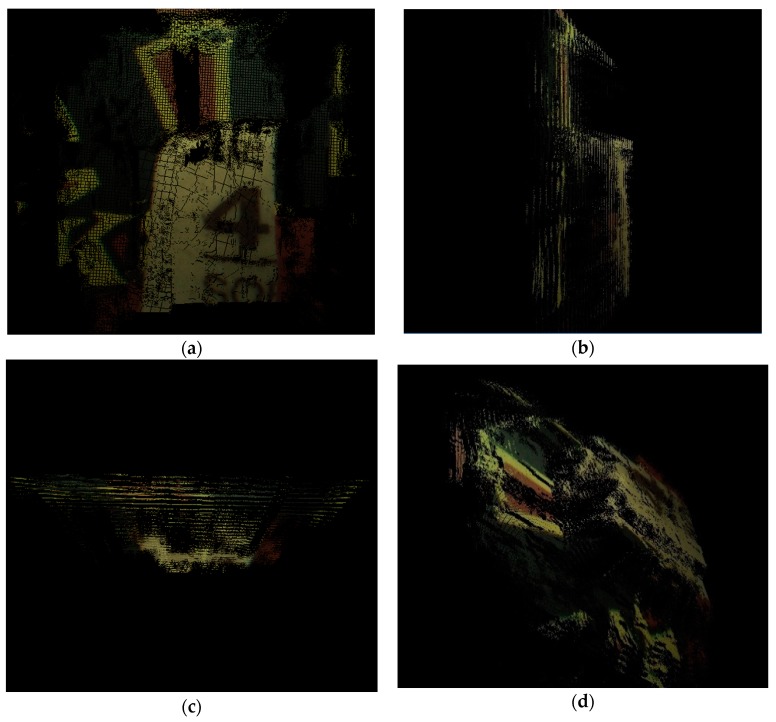
(**a**) The front view; (**b**) The left view; (**c**) The top view; (**d**) An arbitrary view.

**Figure 19:**
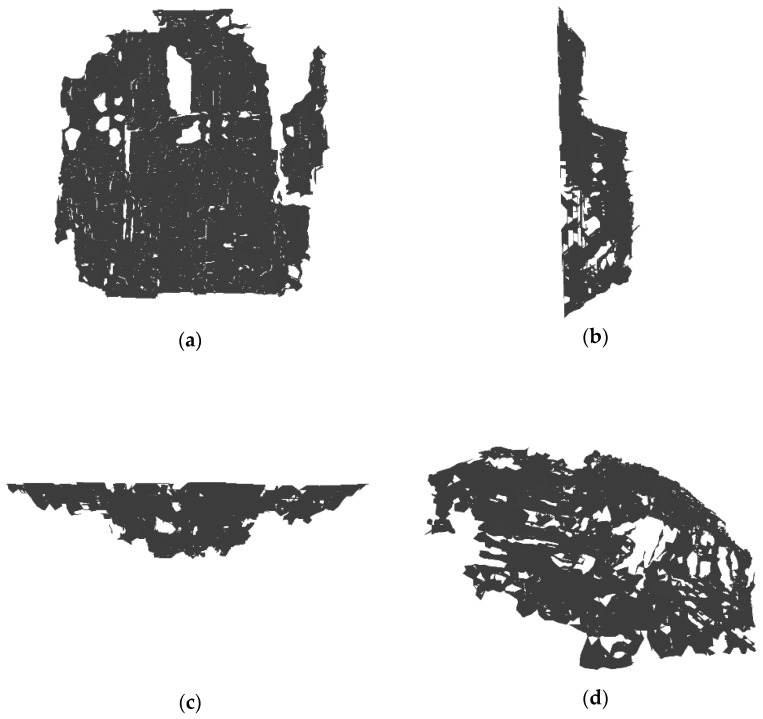
(**a**) The front view; (**b**) The left view; (**c**) The top view; (**d**) An arbitrary view.

The proposed stereo matching algorithm was utilized for 3D reconstruction of the images taken by the endoscope. The endoscope took two images from the two cameras respectively in the distance of about 2 cm and the rectified images are shown in Figure 17. The area of the real scene in every image is about 25 cm^2^. The proposed stereo matching algorithm was applied in the images to obtain a disparity map. Then 3D point clouds data of the left image can be deduced by Equation (3) with the known f and B and they are shown in Figure 18 with PCL. Besides, 3D reconstruction for the real scene which is shown in Figure 19 was achieved with an open source system named MeshLab. In order to present the results spatially, we display them on the font view, the left view, the top view and an arbitrary view.

## 4. Discussion and Conclusions 

A prototype of a miniaturized endoscope, a novel local feature descriptor and a stereo matching algorithm were presented in this paper. The endoscope is based on the principle of binocular stereo vision and its probe has a maximum diameter of 3.17 mm which gives it the ability to access confined spaces for 3D measurement and 3D reconstruction. The endoscope can match POI accurately by the proposed WOS-LBP descriptor and calculates their 3D coordinates by triangulation to measure the sizes and positions of objects. Pairs of images taken by the endoscope can be utilized for 3D reconstruction by the proposed stereo matching algorithm. Experiments on length measurement and 3D reconstruction show that the endoscope can be used to measure and reconstruct real scenes effectively. In general, the designed endoscope exhibits several advantages and potential for industrial and medical measurement.

## Figures and Tables

**Table 1:** The average construction times of the five descriptors.

Descriptor (Size)	WOS-LBP (120)	CS-LBP (240)	LBP (3840)	SIFT (128)	SURF (64)
time/ms	0.6434	0.6588	1.1427	3.3605	0.0750

**Table 2:** Test results of robustness to noise.

Noise Type	Average Matching Rate/%				
	WOS-LBP	CS-LBP	LBP	SIFT	SURF
No noise	99.88	99.88	98.63	100.00	100.00
Gaussian noise	2.63	2.25	1.63	31.37	40.13
Salt-and-pepper noise	60.63	62.25	75.13	38.00	46.38
Average	54.38	54.79	58.46	56.47	62.17

**Table 3:** Parameter values.

	The Parameters Used in Experiment													
Parameters	σ	$\gamma_{census}$	$\gamma_{AD}$	$\tau_{1}$	$\tau_{2}$	$L_{1}$	$L_{2}$	$\beta_{1}$	$\beta_{2}$	$\tau_{1}^{\prime }$	$\tau_{2}^{\prime }$	$L_{1}^{\prime }$	$L_{2}^{\prime }$	Th
Values	1.5	25	30	20	10	15	30	50	500	15	7.5	7.5	15	1

**Table 4:** Percentage of bad matching pixels.

Algorithm	Avg	Tsukuba			Venus			Teddy			Cones		
		Non	All	Disc	Non	All	Disc	Non	All	Disc	Non	All	Disc
Proposed	8.48	3.00	4.42	9.75	1.00	2.46	7.43	6.00	13.71	15.55	4.00	11.46	11.00
ESAW	8.21	1.92	2.45	9.66	1.03	1.65	6.89	8.48	14.20	18.70	6.56	12.70	14.40
SemiGlob	7.50	3.26	3.96	12.80	1.00	1.57	11.30	6.02	12.20	16.30	3.06	9.75	8.90
SNCC	9.41	5.17	6.08	21.70	0.95	1.73	12.00	8.04	11.10	22.90	3.59	9.02	10.70
GC+occ	8.26	1.19	2.01	6.24	1.64	2.19	6.75	11.20	17.40	19.80	5.36	12.40	13.00

**Table 5:** Results of camera calibration.

	Left Camera	Right Camera
Intrinsic matrix	$\left[\begin{array}{ccc} 252.0886 & 0.2324 & 203.9504\\ 0 & 252.2646 & 199.0243\\ 0 & 0 & 1 \end{array}\right]$	$\left[\begin{array}{ccc} 252.2268 & 0.1189 & 206.0211\\ 0 & 252.6249 & 204.0183\\ 0 & 0 & 1 \end{array}\right]$
Distortion coefficients	(−0.0574, −0.2928, −0.0018, 0.0021, 0.2151)	(−0.0536, −0.3176, −0.0011, −0.0023, 0.2517)
Rotation matrix	$\left[\begin{array}{ccc} 0.9962 & 0.0031 & -0.0876\\ 0.0004 & 0.9992 & 0.0394\\ 0.0876 & -0.0393 & 0.9954 \end{array}\right]$	
Translation vector	${\left[\begin{array}{ccc} -1.5976 & 0.1294 & -0.0494 \end{array}\right]}^{T}$	

**Table 6:** Test results of length measurement.

Experiment	Results/mm	Error/mm	Depth/mm	Relative Error/%
1	0.9971	0.0029	2.76	0.29
2	0.9458	0.0542	2.50	5.42
3	0.9199	0.0801	3.53	8.01
4	0.9965	0.0035	3.35	0.35
5	0.9360	0.0640	5.55	6.40
6	0.9956	0.0044	4.60	0.44
7	0.9850	0.0150	5.23	1.50
8	0.9780	0.0220	3.45	2.22
9	0.9560	0.0440	2.55	4.44
Average	0.9678	0.0322	3.72	3.22
